# The law code of ChatGPT and artificial intelligence—how to shield plastic surgeons and reconstructive surgeons against Justitia's sword

**DOI:** 10.3389/fsurg.2024.1390684

**Published:** 2024-07-26

**Authors:** Leonard Knoedler, Alexander Vogt, Michael Alfertshofer, Justin M. Camacho, Daniel Najafali, Andreas Kehrer, Lukas Prantl, Jasper Iske, Jillian Dean, Simon Hoefer, Christoph Knoedler, Samuel Knoedler

**Affiliations:** ^1^ Department of Plastic, Hand, and Reconstructive Surgery, University Hospital Regensburg, Regensburg, Germany; ^2^ Corporate/M&A Department, Dentons Europe (Germany) GmbH & Co. KG, Munich, Germany; ^3^ UC Law San Francisco (Formerly UC Hastings), San Francisco, CA, United States; ^4^ Division of Hand, Plastic and Aesthetic Surgery, Ludwig-Maximilians-University Munich, Munich, Germany; ^5^ College of Medicine, Drexel University, Philadelphia, PA, United States; ^6^ Carle Illinois College of Medicine, University of Illinois Urbana-Champaign, Urbana, IL, United States; ^7^ Department of Cardiothoracic and Vascular Surgery, Deutsches Herzzentrum der Charité (DHZC), Berlin, Germany; ^8^ School of Medicine, University of Pittsburgh, Pittsburgh, PA, United States; ^9^ Morgan, Lewis & Bockius LLP, Munich, Germany; ^10^ Faculty of Applied Social and Health Sciences, Regensburg University of Applied Sciences, Regensburg, Germany

**Keywords:** ChatGPT, artificial intelligence, AI, chatbots, law, legal, lawsuits, litigation

## Abstract

Large Language Models (LLMs) like ChatGPT 4 (OpenAI), Claude 2 (Anthropic), and Llama 2 (Meta AI) have emerged as novel technologies to integrate artificial intelligence (AI) into everyday work. LLMs in particular, and AI in general, carry infinite potential to streamline clinical workflows, outsource resource-intensive tasks, and disburden the healthcare system. While a plethora of trials is elucidating the untapped capabilities of this technology, the sheer pace of scientific progress also takes its toll. Legal guidelines hold a key role in regulating upcoming technologies, safeguarding patients, and determining individual and institutional liabilities. To date, there is a paucity of research work delineating the legal regulations of Language Models and AI for clinical scenarios in plastic and reconstructive surgery. This knowledge gap poses the risk of lawsuits and penalties against plastic surgeons. Thus, we aim to provide the first overview of legal guidelines and pitfalls of LLMs and AI for plastic surgeons. Our analysis encompasses models like ChatGPT, Claude 2, and Llama 2, among others, regardless of their closed or open-source nature. Ultimately, this line of research may help clarify the legal responsibilities of plastic surgeons and seamlessly integrate such cutting-edge technologies into the field of PRS.

## Introduction

Artificial Intelligence (AI) has witnessed remarkable advancements in recent years, revolutionizing various sectors, including medicine. The integration of AI into healthcare holds immense potential to enhance diagnostic capacity, treatment, and overall patient care (1). AI, encompassing machine learning, natural language processing, and data analytics, has emerged as a valuable tool in medicine. AI algorithms can analyze vast amounts of medical data, detect patterns, and generate insights that assist in diagnosis, treatment planning, and patient monitoring (2–4).

Large Language Models (LLMs) like ChatGPT 4 (OpenAI), Claude 2 (Anthropic), and Llama 2 (Meta AI) represent the most recent use case of AI leveraging natural language processing to autonomously respond to questions and complete tasks (5). For healthcare providers, LLMs have been proposed as valuable tools for interpreting laboratory values, generating novel research ideas, and advancing patient education (6, 7). Overall, AI in general, and LLMs in particular carry the potential to disburden the healthcare system and improve patient care. While numerous trials are elucidating the untapped potential of AI tools for use cases in plastic and reconstructive surgery (PRS), the sheer speed of scientific progress takes its toll, too.

To date, there is a paucity of studies that clarify the legal guidelines when using Large Language Models and AI based tools for PRS scenarios. This knowledge gap poses the jeopardy of lawsuits and penalties against PRS institutions (academic and non-academic hospitals; medical healthcare centers; outpatient surgical centers) and plastic surgeons who already face a 15% risk per year of being sued (8, 9). However, this scarcity of studies affects the entire field of medical healthcare.

Herein, we aim to provide the first summary of US legal guidelines for implementing Large Langue Models like ChatGPT and other AI based tools into PRS everyday work. Ultimately, this line of research may provide a robust legal foundation to facilitate clinical AI use and position PRS at the pole-position of future AI research.

## Prescription of drugs or treatments using large language models and other AI-based tools: legal considerations for surgeons

In cases of PRS malpractice, surgeons breach their legal obligations when they fail to meet the standard of care. As of now, there have been no specific court cases directly addressing liability related to the use of LLMs in PRS, primarily due to the novelty of the technology and its ongoing implementation. Consequently, the subsequent analysis is based on the broader application of medical malpractice law.

Elements of PRS malpractice include breach, causation, and damages. At base, physicians have a duty to treat their patients according to the standard of care. The standard of care is understood as the care that would be provided by a competent physician of the same specialty, taking into consideration the resources that are available at the time of patient treatment and/or consultation (10–13). The interplay between artificial intelligence and the standard of care is intricate and expected to evolve over time. Plastic surgeons may face legal consequences when recommending surgical procedures or treatments using ChatGPT or comparable models. A significant concern in using LLMs in healthcare is not only the potential for professionals to misinterpret the model's information but also the risk of LLMs suggesting and disseminating misguided and unreliable surgical or treatment recommendations for patients. Under the current legal framework, a plastic surgeon can be held fully liable in medical malpractice cases when relying on suggestions from a language model.

As a concrete example, consider a plastic surgeon treating a patient for a facial reconstructive procedure. The standard of care is procedure X, a surgical method with known moderate side effects. Another procedure, procedure Y, is approved for non-reconstructive use in trauma patients, but observational studies have shown that it may enhance facial reconstruction significantly. However, procedure Y, displays a potentially higher complication profile and is therefore discouraged for use in facial reconstruction. The surgeon will choose one procedure or the other. The surgeon enters their patient's information into the electronic health record, and an embedded AI system makes a recommendation for a given surgical procedure (10, 14, 15).

Since malpractice law commonly absolves liability for adhering to the standard of care, clients would generally not be justified in blaming surgeons for AI tool usage that aligns with the standard of care, even if the outcome is suboptimal. If the procedure yields positive results, there is clearly no liability since there is no injury. Even if the procedure proves ineffective, surgeons usually remain shielded from liability as long as their actions align with the accepted standard of care.

On the other hand, if a surgeon follows AI's suggestion and performs a procedure that deviates from the standard of care, the likelihood of liability increases. If the chosen procedure is suitable for the patient and no harm occurs, then there is no liability. However, if the chosen procedure is unsuitable for the patient and injury ensues, the surgeon is likely to be held liable for actions that fall below the standard of care, regardless of AI's recommendations. The treating surgeon cannot then exculpate themselves by relying on the recommendation's compliance with the standard of care.

Another point to keep in mind is that the legal standard of care is not static and continually evolves. With the emergence of AI-powered technologies, these advancements may eventually become part of the “standard of care” in PRS. Therefore, it is conceivable that late adapters may risk violating the standard of care if they fail to adopt evidence-based beneficial AI that most other doctors have already accepted and integrated into patient care (12).

Some observers in the field of PRS have highlighted that the current medical malpractice law creates incentives for surgeons to downplay the potential benefits of AI. They argue that, to mitigate liability risks, the safest approach for surgeons is to utilize AI primarily as a “confirmatory tool” that supports existing decision-making processes, rather than viewing it to enhance care (13). In fact, a current study shows that physicians currently may use AI technology the most in “low uncertainty” cases, when they are pretty sure of a prospective treatment plan but avoid using it in higher-uncertainty cases (16).

As AI becomes an integral part of healthcare, it is crucial to address its role in patient consent processes. Discussions about whether patients want AI used in their assessment and treatment need to be incorporated into the routine informed consent process. Even though the patient has consented to a proposed treatment or operation, the failure of the physician to inform the patient adequately before obtaining such consent is negligence and renders the physician subject to liability for any injury resulting from the treatment or operation. Generally, the question of whether information must be disclosed is centered on whether the information would be considered significant by a reasonable person in the patient's position when deciding to accept or reject a recommended medical procedure (17). Following this general guideline, a physician does have a duty to disclose the use of AI-based tools and its extent if the information is material to the patient's decision-making. To be protected from liability and fully respect patient autonomy, hospitals should explicitly mention in their general consent to treatment forms whether and to what extent they use AI assisted tools. More specifically, physicians should explain how these tools contribute to their recommendations. Another important consideration is how much detail physicians need to disclose about using AI tools. Specifically, physicians may wonder if they must explain how the AI tool arrived at its conclusions, the workings of the algorithm, and the data it was trained on. Without specific case law, this question can only be addressed based on current standards. Generally, doctors are not required to explain their entire thought process or the quality of sources they consulted for their decisions. Similarly, detailed explanations of the AI model's inner workings and training data are not usually necessary. However, any known biases in the data that could affect the tool's recommendations should be communicated to the patient.

The utilization of LLMs can lead to further legal risks. For instance, using ChatGPT may result in legal infringements related to the processing of personal data (18). Healthcare providers bear significant responsibility for ensuring AI technologies are deployed ethically and in adherence with regulations protecting patient data confidentiality. In the US, the main federal law governing the privacy and protection of health-related personal data is the Health Insurance Portability and Accountability Act (HIPAA). Under HIPAA guidelines, if covered healthcare providers use AI tools for treatment and discloses patient information in the process, they are required to provide a Notice of Privacy Practices (NPP) to the patient, informing them of this potential use and disclosure of their information (19). Additionally, covered healthcare providers must obtain the individual's written authorization for any use or disclosure of protected health information that is not for treatment, payment, health care operations or otherwise permitted or required by the Privacy Rule (20). It is important to note that the transfer of patient information to ChatGPT or any other chatbot is generally not exempt from this rule. Given that the definition of “treatment” includes the coordination and management of health care services, as well as consultations related to patient care (21), it is conceivable that the exemption under HIPAA for obtaining consent extends to the use of AI tools and chatbots for treatment purposes. However, in the absence of current legislative guidelines, it is advisable to obtain proper consent for the transfer of patient data and to be transparent about how AI is being employed in providing care.

An alternative approach to potentially bypass these regulations involves de-identifying the data before interacting with a language model (22). If the patient information is covered by HIPAA, de-identification of the protected health information (PHI) requires the removal of certain identifiers or an expert's determination that data can be considered de-identified (23). Some observers, however, note that this method does not provide conclusive proof against subsequent re-identification (18). To ensure the highest level of safety, surgeons should refrain from inputting any content into such tools that might contain a patient's personal data, confidential information, or any data that is not meant to be disclosed to third parties.

In the great scheme of liability, it is important to recognize that if ChatGPT provides misleading or inaccurate information, there may not be sufficient grounds to claim against OpenAI. Their terms of service clearly state that liability is excluded to the fullest extent possible. This applies to both product liability claims from patients and indemnification claims from physicians using the AI tool for treatment. Currently, there are no legal precedents on whether these liability limitations for AI use in medical treatment are enforceable. Therefore, in the absence of clear judicial guidance, these limitations should be considered enforceable.

## Institutional liability for use of artificial intelligence in plastic and reconstructive surgery

Under which circumstances can hospitals be held liable for adverse events caused by the use of AI (e.g., ChatGPT) during a PRS procedure? One must distinguish two separate theories—derivative liability for the actions of plastic surgeons or others and direct liability for the institution itself.

Derivative liability first requires proving medical malpractice or another form of liability by the plastic surgeon or healthcare provider. Once malpractice is established, legal theories connect this liability to the hospital. Without proven malpractice, the hospital's liability is excluded. The conditions under which medical malpractice liability may be imposed vary based on whether the plastic surgeon is a hospital employee or an independent contractor. Under the doctrine of respondeat superior, employers are liable for employees' actions within the scope of their employment. If a hospital employee misuses AI, such as ChatGPT, resulting in malpractice, the hospital may be liable. This principle extends to those under significant hospital control, even if not formally employed (23).

For independent contractors, the respondeat superior theory does not apply. Instead, liability may arise under the “apparent authority” doctrine. Apparent authority occurs when a third party reasonably believes an individual has authority to act on behalf of another party, based on the principal's representation. To establish apparent authority, two conditions must be met: the principal must have represented the agent as having authority or knowingly allowed the agent to act on its behalf, and the plaintiff must have relied on these representations. In the context of AI in PRS, if an independent contractor plastic surgeon misuses AI and causes harm, and the hospital has presented the surgeon as its agent, leading the patient to reasonably rely on this representation, the hospital may be liable.

Hospitals also bear responsibilities towards their patients that can give rise to direct liability for the institution itself. These legal theories are relevant to the decisions hospitals make regarding the implementation of AI in PRS. Even though there have been no reported decisions yet specifically addressing such scenarios, there are two main hospital direct liability theories that courts may apply to the use of AI in PRS in the future and that can lead to hospitals' direct liability: (i) negligent selection/retention and (ii) negligent supervision.

The theory of negligent selection and retention imposes upon a hospital system a duty to review “surgeons” competency and performance history before admission to the medical staff and periodically thereafter” (24). In order to recover a plaintiff must show that the hospital did not exercise reasonable care, meaning the care ordinarily exercised by the average hospital, to determine whether the surgeon was competent (25). From a plaintiff's standpoint, it could be argued that a hospital system is essentially engaging in a hiring process rather than a simple purchase when acquiring an AI system. Consequently, this would impose responsibilities on the hospital system to assess previous errors resulting in adverse events linked to the use of the AI, review the certification process, ascertain the individuals involved in the certification, evaluate the quality of certification, and potentially determine how the AI system will integrate with the existing hospital workforce, similar to the considerations made when hiring a human surgeon. Courts may deem this theory as going too far in terms of attributing human-like characteristics to AI systems. Even if the theory is endorsed, the practical assessment of negligence relies, to some extent, on comparing the level of care employed in these determinations with that practiced by other hospital systems. This presents a complication, particularly during the initial stages of AI integration in healthcare, where establishing a standard for care becomes problematic (25).

The theory of negligent supervision on the other hand operates under the assumption that the duty lies in the contemporaneous supervision of surgical decisions “as they are being made” (24). In the future, courts may impose a duty upon hospitals to supervise each AI recommendation and/or reliance thereon by a plastic surgeon “as they are made” in addition to the negligent selection/retention duties and whatever derivative liability exists. Although there have been references to such a duty in several rulings, it has predominantly been imposed in cases involving “gross negligence, in which the departure from medical standards is so blatant that it is possible to attribute to hospital administrators’ constructive knowledge of the error in progress” (24). Some observers believe that courts will be more skeptical of negligent supervision theories, especially when it comes to more opaque forms of AI in PRS (26).

Supplementary Material S1 shows a checklist to be used by plastic surgeons and medical academic institutions when implementing AI and chatbots.

## Conclusion

**Figure 1:**
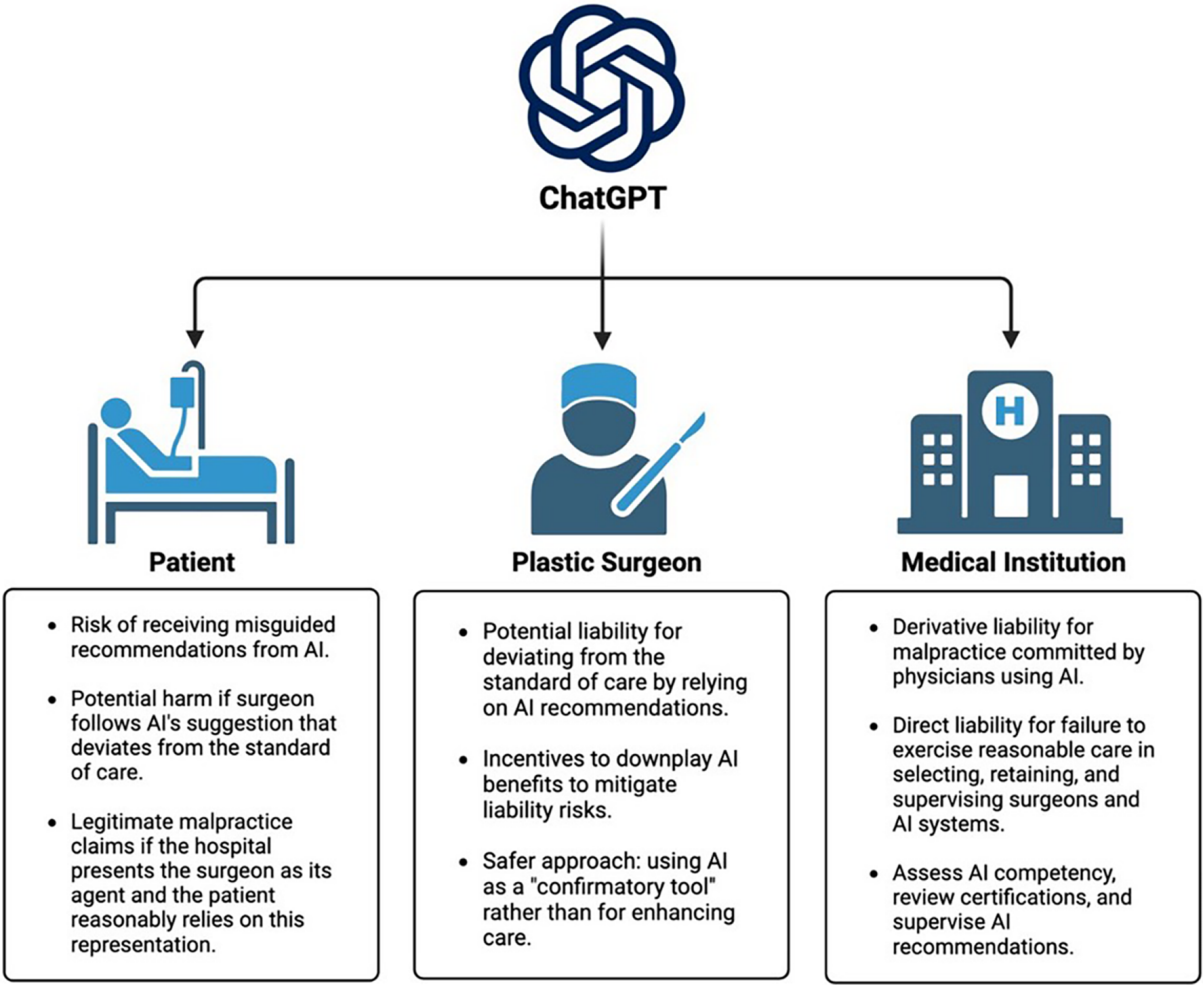
Multi-level legal aspects of artificial intelligence and ChatGPT.

In conclusion, the current liability framework for physicians and hospitals regarding the use of AI systems such as ChatGPT in PRS is based on general principles of malpractice liability (Figure 1). However, there is significant uncertainty surrounding how these factors will be interpreted as cases begin to reach the courts. Additionally, legislative, and regulatory interventions could potentially bring about substantial changes in this landscape. Notably, the standard of care is expected to evolve as the use of AI becomes more widely accepted in PRS. However, the pace of adoption is likely to vary across different areas of surgical practice. As we navigate the dynamic intersection of AI and plastic surgery, it is crucial to closely monitor legal developments and anticipate the ongoing evolution of standards and regulations in PRS. Ultimately, these research efforts may place PRS at the forefront of evidence-based and law-compliant AI research.

## Data Availability

The original contributions presented in the study are included in the article/**Supplementary Material**, further inquiries can be directed to the corresponding author.

## Supplementary material

Supplementary Material S1 Checklist for the law-compliant use of artificial intelligence and chatbots.
