# Supplementary material for: The law code of ChatGPT and artificial intelligence—how to shield plastic surgeons and reconstructive surgeons against Justitia's sword
Source: Front Surg. 2024 Jul 26;11:1390684. doi: 10.3389/fsurg.2024.1390684 (PMC11312379; doi:10.3389/fsurg.2024.1390684)
Supplement: Supplementary Material S1 — Checklist for the law-compliant use of artificial intelligence and chatbots. [file Datasheet1.pdf]

- Before implementing a procedure recommended by artificial intelligence (AI), the treating surgeon must verify that it aligns with the established standard of care. If the chosen treatment is unsuitable for the patient and injury occurs, the surgeon is likely to be held liable for actions that fall below the standard of care, regardless of ChatGPT's or any other chatbot's recommendations.

- As the standard of care is subject to constant change, surgeons should stay attentive in monitoring legal advancements and remain receptive to implementing cutting-edge AI systems. As AI-powered technologies continue to emerge, it is likely that these advancements will soon become integral components of the standard of care. In case of evidence-based beneficial AI already implemented by most healthcare providers, it is conceivable that delayed adaptations may violate the standard of care.

- To ensure compliance with data protection regulations and prevent any legal violations, surgeons should refrain from inputting any information in ChatGPT that might contain a patient's personal data, confidential information, or any other data that is not meant to be disclosed to third parties.

- Medical institutions should be aware of the potential risk of derivative liability for medical malpractice that may arise from the actions of their surgeons utilizing AI. Consequently, it is also within the hospital's best interest to monitor legal advancements, establish internal guidelines for working with AI, and provide regular training to its staff on the utilization of AI-supported applications.
